# In vitro study of the apical microleakage with resilon root canal filling using different final endodontic irrigants

**DOI:** 10.4317/jced.51755

**Published:** 2015-04-01

**Authors:** Eduard Lahor-Soler, Jaume Miranda-Rius, Lluís Brunet-Llobet, Magí Farré, Josep Pumarola

**Affiliations:** 1 DDS, Endodontics Unit. Departament d’Odontostomatologia. Facultat d’Odontologia. Universitat de Barcelona, Barcelona, Spain; 2 MD, DDS, PhD, Periodontics Unit. Departament d’Odontostomatologia. Facultat d’Odontologia. Universitat de Barcelona, Barcelona, Spain; 3 MD, DDS, PhD, Paediatric Dentistry and Orthodontics Unit. Servei d’Odontologia. Hospital Universitari Sant Joan de Déu. Universitat de Barcelona, Barcelona, Spain; 4 MD, PhD, Clinical Pharmacology Unit. Hospital Universitari Germans Trias i Pujol-IGTP and Hospital del Mar Medical Research Institute-IMIM. Facultat de Medicina. Universitat Autònoma de Barcelona, Barcelona, Spain; 5 MD, DDS, PhD, Endodontics Unit. Departament d’Odontostomatologia. Facultat d’Odontologia. Universitat de Barcelona, Barcelona, Spain

## Abstract

**Background:**

Endodontic microleakage or microfiltration refers to the percolation of fluids and micro-organisms at the interface of the obturation material and the walls of the root canal system. The aim of this in vitro study was to compare apical microfiltration of Resilon root canal filling by employing three different final irrigant solutions.

**Material and Methods:**

128 single-rooted teeth were employed. The crowns were sectioned horizontally at the cemento-enamel junction and instrumented with 5.25% sodium hypochlorite (NaOCl) and 17% EDTA gel to obtain an instrumented 040 apical caliber. An intermediate irrigation was performed with distilled water. The roots were then randomly assigned to three experimental groups with three different final irrigants: (A) 20% citric acid (CA); (B) 2% chlorhexidine digluconate (CHX); and (C) 5.25% NaOCl, plus two control groups (positive and negative). They were then dried, obturated with RealSeal™, and cleared by Robertson’s technique. Apical microleakage was measured by the dye penetration method and assessed with a 4.5x stereomicroscope. Data were statistically analyzed by one way ANOVA and post hoc analysis for multiple comparisons.

**Results:**

Mean and standard deviations for apical microleakage were: 2% CHX (0.24 mm ± 0.22), 20% CA (0.25 mm ± 0.20), and 5.25% NaOCl (0.87 mm ± 0.32). Significant differences were reported among the group irrigated with NaOCl, CHX and CA (P<0.001).

**Conclusions:**

A higher rate of apical microleakage was observed when the final irrigation was performed with NaOCl whilst lower rates were reported for CHX and CA.

** Key words:**Apical filtration, endodontic irrigation, resin-based sealers, adhesion, root canal filling.

## Introduction

Chemo-mechanical preparation of the root canal system makes intimate contact possible between the intertubular and peritubular dentine and the obturation material (1). Irrigants are used during the procedure not only as antimicrobial agents, but also to lubricate the dentinal walls, remove debris, and dissolve organic and inorganic components of the smear layer (2).

Endodontic microleakage or microfiltration refers to the percolation of fluids and micro-organisms at the interface of the obturation material and the walls of the root canal system, and also through the existing gaps in the obturation material itself.

Both coronal and apical microleakage have an adverse effect on the outcomes of root canal treatment. The use of products with adhesive properties such as root canal sealers has been investigated in order to obtain a better chemical bond between the dentine and the core material. Various study groups have observed significantly less coronal and apical microfiltration in canal obturations where dentine adhesive agents, sealers, and gutta-percha have been employed than in those where only sealing material and gutta-percha were used (3,4).

Resilon™ (Resilon Research LLC, Madison CT, USA), which has similar handling properties to gutta-percha, is a root canal filling material made up of synthetic polyester polymers, bioactive glass, and radiopaque filling. It is used with its sealer which has two components: a self-etch primer and dual-cure resin-based sealer. This obturation system is marketed as RealSeal™ (SybronEndo, Orange, CA, USA) or Epiphany™ (Pentron Clinical Technologies, Wallingford CT, USA). A chemical bond is, therefore, obtained with the filling material, sealing agent, and dentine walls forming the Resilon monoblock system (RMS) (5,6). The term monoblock, literally meaning a single unit, has generated controversial discussion in endodontics as to whether it is able to improve seal quality in root fillings and strengthen roots. RMS is a secondary monoblock with two circumferential interfaces, one between the cement and dentin, the other between the cement and the core material (7).

The quality and strength of the dentine adhesive bonds are greatly affected by treatment previously carried out on the dentine walls. Smear layer removal, collagen fibril alteration, and the level of exposure of the dentinal tubules are determining factors (8,9). With respect to irrigants, chlorhexidine digluconate (CHX) displays antimicrobial action, firmness, and adequate biocompa-tibility (10-12). Sodium hypochlorite (NaOCl), which also has antimicrobial properties and the capacity to disrupt biofilms, is, however, the only irrigant capable of dissolving organic tissue (10,13,14). As neither NaOCl nor CHX have the capacity to dis-solve inorganic particles or eliminate the smear layer, it is necessary to introduce acids or chelating agents such as citric acid (CA) which is able to remove tooth smear layer without causing any changes in the collagen fibers. Another irrigant that has properties very similar to CA is ethylenediaminetetraacetic acid (EDTA) although it appears to have a lesser effect at the same concentration (10,15).

The purpose of this study was to compare the amount of apical leakage occurring with RealSeal root canal filling using three different final irrigant solutions: 20% CA; 2% CHX; and 5.25% NaOCl.

## Material and Methods

An *in vitro* experimental study was designed with a sample composed of 128 extracted single-root canal teeth conserved in sterile distilled water. All the samples had a mature apical foramen, intact root surface, and root length ≥ 10 mm. They had not previously undergone root canal treatment and were without calcifications as verified by the patency of the canals.

The root surfaces had been cleaned with Columbia 4R / 4L periodontal curettes (Hu-Friedy, Chicago, IL) and an ultrasonic scaler (Acteon-Satelec P5 Booster, Cedex, France). A 4.5x stereomicroscope (Olympus SZ 61 Hamburg, Germany) was employed to rule out any possible external radicular defects.

The 128 teeth were randomly assigned to three experimental groups (n=40) according to the final irrigant used and two controls, one negative (n=4) and another positive (n=4). The crowns were horizontally sectioned at the cemento-enamel junction with a Struers Accutom-5 (Struers A/S, Rodovre, Denmark) and the root canals instrumented with size 10 K-files (Dentsply/Maillefer, Ballaigues, Switzerland). Working length (WL) was calculated by placing the K-file into the root canal until visible at the apical foramen and subtracting 1mm. A glide path was performed with K-files to a caliber of 025 from the WL and step-backs of 1mm to a caliber of 035. Next, the coronal two-thirds of the canals were shaped with a K3 file rotary system (SybronEndo SDS, Orange, CA, USA) in the following sequence: 25/.12, 25/.10, 25/.08 and the apical third to 35/.06 at WL. Apical caliber was standardized to 040 with step-backs of 0.5 mm using LS1 Lightspeed files (LightSpeed Technology Inc, San Antonio, TX). Apical patency was maintained by passing a 10 K-file through the apical foramen.

During instrumentation, irrigation was carried out with 6ml of 5.25% NaOCl using an endodontic irrigation syringe with a Monoject™ side hole needle (Kendall-Tyco Healthcare group, Mansfield, MA). 17% EDTA gel (Glyde, Dentsply, Surrey, United Kingdom) was employed as a chelating agent. Between the irrigations of 5.25% NaOCl and the final one, an intermediate irriga-tion was performed with 10 ml of distilled water (DW) to remove the chemical auxiliary substance.

The final irrigation for each experimental group was performed with aqueous solutions containing CA, CHX, and NaOCl. Group A: 20% CA 6 ml; group B: 2% CHX 6 ml; and group C: 5.25% NaOCl 6 ml. In all three groups the final irrigant solution was allowed to remain for 60 seconds inside the root canals in order to permit greater activity on the dentine tissue.

Afterwards the canals were dried with paper points, RealSeal primer was applied using the kit applicator and left to act for 25 seconds, any excess was removed with a paper point. A sealer was applied anti-clockwise using a 40 K-file and a Resilon 40/.04 point introduced at WL. Lateral condensation was carried out with X-fine Resilon accessory points and a 25 digital spacer until root obturation. Excess was cut 1mm below the root entrance with a heated plugger and the entrance light-cured for 40 seconds.

**Figure 1:**
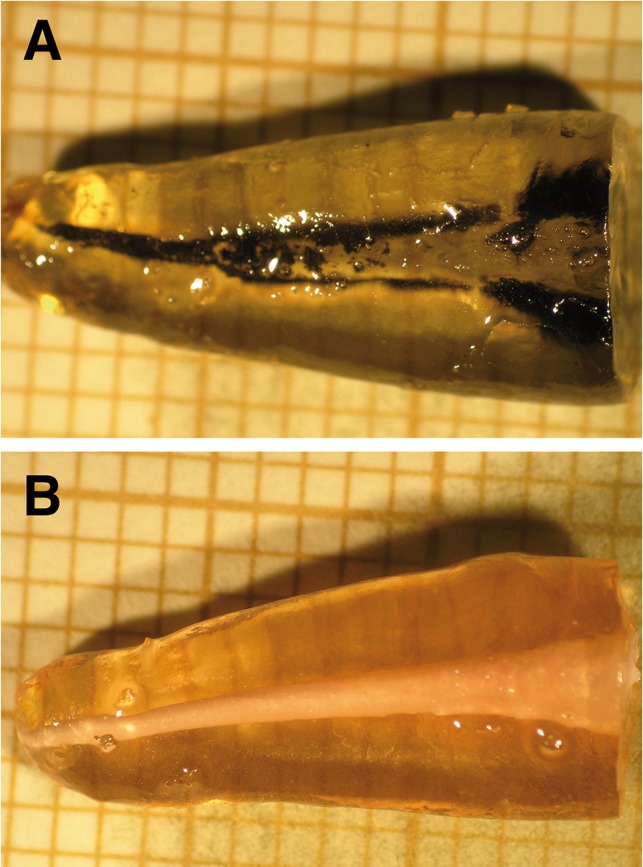
A) Positive Control: In the absence of obturation, note how the Indian ink shows maximum filtration along the root canal. Original magnification 4.5x. B) Negative Control: With obturation and one impermeable varnish layer, observe how the Indian ink does not infiltrate anywhere along the root canal. Original magnification 4.5x.

Samples were stored for three days. In order to simulate intra-oral conditions of humidity and temperature they were submerged in distilled water at 37ºC. In a next step, using the dye penetration method, the apical 3 mm of each tooth was submerged in 2 ml of Pelikan™ Indian Ink (Pelikan, Hannover, Germany) for seven days. To verify the correct functioning of the Indian ink immersion, two control groups were established: one positive, with leakage; and the other negative or leakage-free (Fig. 1). Teeth in the positive control group (n=4) were prepared in the same way as groups A, B, and C but without obturation of the root canals; in the negative group (n=4) preparation and obturation was the same as groups A, B, and C but, on finishing, a coat of resin-based sealing varnish was applied to all the exterior surfaces of the roots to ensure impermeability.

After immersion in Indian ink the samples were washed with distilled water for two days. They were then cleared following the technique described by Robertson *et al.*, (16) which involves decalcification of the samples with 5% nitric acid for three days, dehydration with increasing concentrations of alcohol (80-100%), and transparency with methyl salicylate.

**Figure 2:**
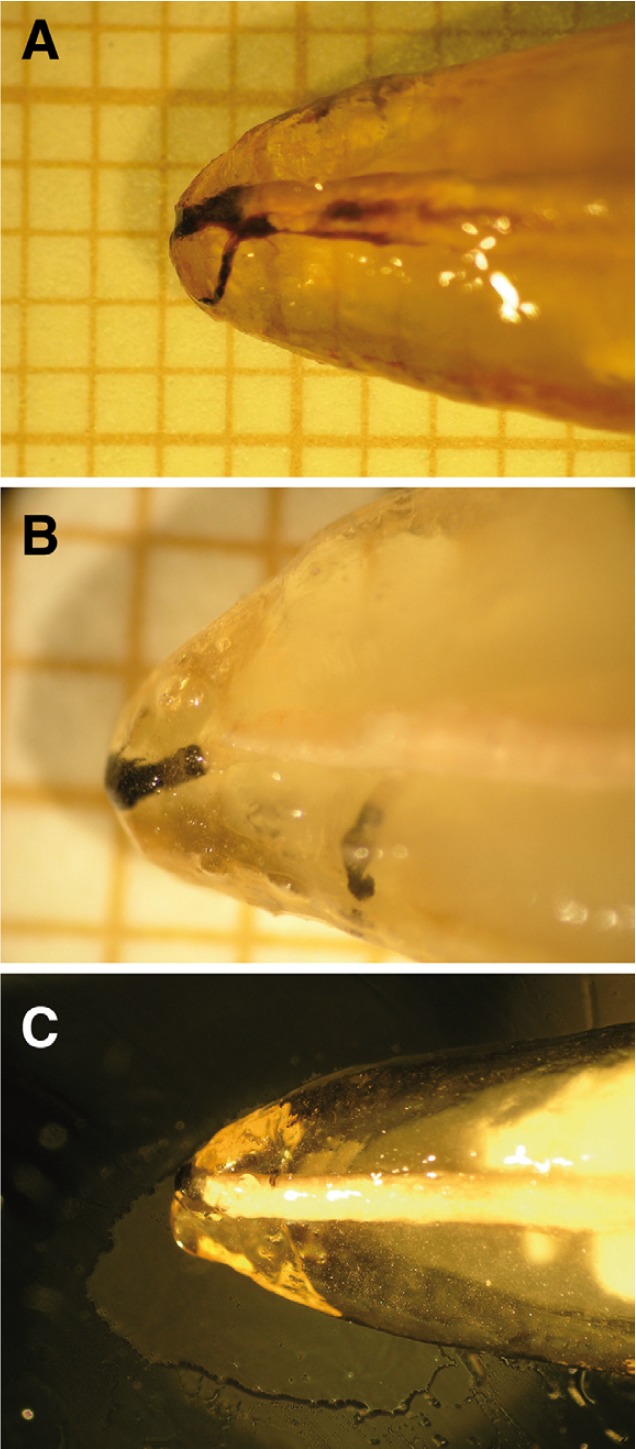
A) Sodium hypochlorite group: High apical filtration in this experimental group. Original magnification 4.5x. B) Chlorhexidine digluconate group: Minimum apical filtration in this experimental group. Original magnification 4.5x. C) Citric acid group: Low apical filtration in this experimental group. Original magnification 4.5x.

All samples were prepared by the same researcher. The blinded assessment was performed by two trained researchers. The apical microleakage was registered in millimeters and both examiners agreed on the final value. The measurement was performed with the help of 4.5x stereomicroscope and millimetric paper. The four faces of each tooth were measured, and the largest microleakage measurement was registered and photographed (Olympus Camedia C-5060 WideZoom, Tokio Japan) (Fig. 2).

Data were analyzed by one way ANOVA and post hoc multiple comparisons using the Bonferroni test to compare apical micro-leakage. Level of significance was set at *P*<0.05.

## Results

Apical microleakage mean values, standard deviations, and confidence intervals of the studied samples are shown in  Table 1.

**Table 1:**
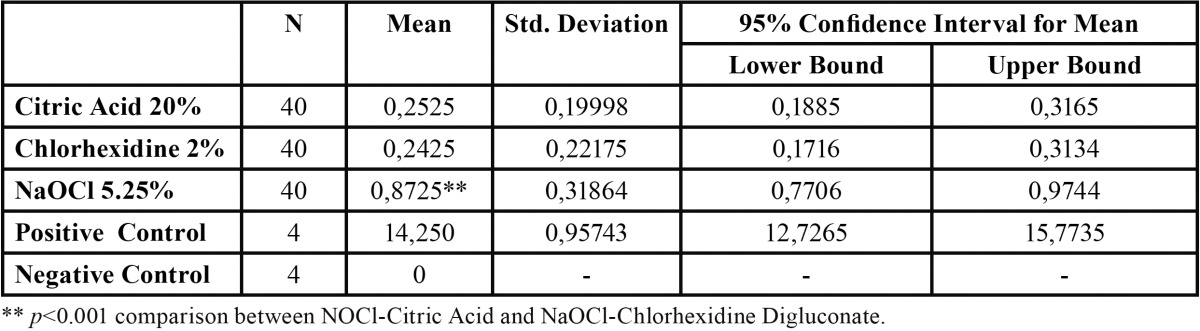
Apical microleakage values (mm) of the experimental and control groups.

With respect to the control groups, maximum filtration values were observed in the positive group as the Indian ink infiltrated all the root canal (14.25 mm ± 0.95) whilst no infiltration was reported for the negative group (0 mm) (Fig. 1).

The lowest apical microleakage values were found in the group which had 2% CHX as its final irrigant (0.24 mm ± 0.22), followed by the group with 20% CA (0.25 mm ± 0.20), although no statistically significant differences were observed between the two groups (*P*=1.00) ( Table 1).

The highest apical microleakage values were reported for 5.25% NaOCl as final flush (0.87 mm ± 0.32) (*P*<0.001).

Significantly high differences were observed between the 5.25% NaOCl group and the 2% CHX (*P*<0.001) and 20% CA groups (*P*<0.001) ( Table 1).

## Discussion

Adhesion is defined as the process by which two surfaces with distinct molecular characteristics bond through physical, chemical or mechanical means (17). In order to provide a stable bond between the obturation material and the root canal walls the adhesion of sealing materials is fundamental and may be influenced by a number of variables which include irrigant solutions (18). Various studies have analyzed solutions such as CHX, EDTA, and NaOCl (6,8,9). In contrast, few have evaluated apical microleakage in Resilon root canal therapy with citric acid as the final irrigant solution.

Results are contradictory concerning both the strength of the bond and the adhesive microfiltration on the dentine walls (6,19-21). RealSeal manufacturer’s instructions recommend EDTA or CHX as final irrigant solutions followed by sterile water flushing as NaOCl and hydrogen peroxide may damage dentine wall adhesion.

In our study, the low values of microleakage observed in the 2% CHX group could be due to the fact that CHX respects the organic dentine matrix. The prior use of EDTA, which eliminates the smear layer, exposes the collagen fibers thus increasing the hydrophilicity of the dentine surface which favors the hybridization of hydrophilic material such as methacrylate-based sealers (14,20,22,23). Reduced leakage is, therefore, caused by greater micromechanical interactions amongst the adhesive agents, the collagen matrix, and the mineralized base of the intertubular dentine (24). A number of studies have observed that a final flush with CHX does not negatively affect bond strength (8,25-27).

The use of 20% CA as a final irrigant in our study was associated with low apical microleakage values. Such values could be justified by its demineralizing capacity and smear layer removal action, both of which favored an increase in exposed dentinal tubules and adequate collagen matrix (10,15). Facilitating the hybrid layer in this way is indispensable for a correct chemical adhesion between the obturation material and the dentine walls (15).

The demineralizing properties of CA can also be found in other chelating agents such as EDTA (10,15). This common characteristic could explain the similar filtration results observed when they are used as final irrigants. Vilanova *et al.* reported that with 1% NaOCl, followed by a final 17% EDTA irrigation, greater adhesive strength was obtained between Epiphany and the root canal walls (9).

In the present study, the elevated leakage values observed in the final flush with 5.25% NaOCl are due to its oxidative capacity. It generates an oxygen-rich layer on the dentine surface which reduces adhesive strength through inhibition of the polymerization of the methacrylate-based resins (28).

Some authors have attributed greater filtration values to the fact that NaOCl acts selectively on the organic components which could explain its incapacity to completely eliminate the amorphous smear layer made up of organic and inorganic detritus. This hinders adequate exposure of the dentinal tubules and deteriorates the collagen fibers, thus preventing a correct hybrid layer formation (10,29). Ozturk & Özer reported that the use of NaOCl produced a significant decrease in bond strength with the dentine walls (19). In a similar manner, Vilanova *et al.* observed that 1% NaOCl as final irrigant produced weaker adhesion (9).

Some studies have mentioned the use of MTAD, a mixture of a tetracycline isomer (doxycycline), an acid (citric acid), and a detergent (Tween 80), as a possible final flush before Resilon obturation. An alternative and more recent substance, MTAD is capable of eliminating both organic and inorganic detritus. Its anti-microbial properties thus permit it to eliminate the smear layer from the root canal surface (30).

Some authors found an improvement in resisting bacterial leakage and fracture resistance of the root canals obturated with Resilon-filled root canals compared with gutta-percha ones (5). Although Resilon-filled root canals do achieve good apical and coronal seals, it is not clear from subsequent independent research studies whether such seals are better than those obtained using gutta-percha and conventional root canal sealers (6,22,24).

In summary, in this in vitro study relevant differences were observed with respect to apical filtration values amongst the distinct irrigant protocols employed. Statistically significant higher microleackeage values from the sodium hypochlorite group in comparison to the other experimental groups were reported. In reference to the two other final irrigants it may be concluded that both chlorhexidine digluconate and citric acid minimize apical microleakage. In order to obtain the correct formation of the Resilon, sealing material, and dentinal wall monoblock it is necessary to bear in mind that it is essential to maintain the collagen matrix structure and carry out a thorough elimination of the smear layer. The use of sodium hypochlorite as a final irrigant should, there-fore, be avoided as its inherent organic material degrading properties can affect the collagen matrix, thus preventing the chemical adhesion of this material in root canal treatment.
